# Professionals’ management of the fidelity–adaptation dilemma in the use of evidence-based interventions—an intervention study

**DOI:** 10.1186/s43058-021-00131-y

**Published:** 2021-03-16

**Authors:** Ulrica von Thiele Schwarz, Fabrizia Giannotta, Margit Neher, Johanna Zetterlund, Henna Hasson

**Affiliations:** 1 School of Health, Care and Social Welfare, Mälardalen University, Box 883, Västerås, Sweden; 2 Procome, Medical Management Centre, Karolinska Institute, Stockholm, Sweden; 3 Department of Rehabilitation, School of Health Sciences, Jönköping University, Jönköping, Sweden; 4 Unit for Implementation and Evaluation, Center for Epidemiology and Community Medicine, Region Stockholm, Sweden

**Keywords:** Professionals, Adaptation, Adherence, Fidelity–adaptation dilemma, Evidence-based interventions, Decision support

## Abstract

**Background:**

Evidence-based interventions (EBIs) can be effective tools for the prevention of disease and health promotion. However, their implementation often requires a delicate balance between the need to adjust the intervention to the context in which it is implemented and the need to keep the core components that make the intervention effective. This so-called dilemma between fidelity and adaptation is often handled by health professionals in the sustainment phase of an implementation (i.e., once the intervention has been adopted and institutionalized in an organization), but not much is known about how and to what extent health professionals are affected by this dilemma. Focusing on the sustainment phase, this project aims to study (1) how fidelity and adaptation are managed by professionals using an EBI, (2) how the fidelity–adaptation dilemma affects professionals’ psychosocial working conditions, and (3) how a structured decision support influences professionals’ management of the dilemma and their psychosocial working conditions.

**Methods:**

The study is set in Sweden, and the EBI in focus is a parental program (All Children in Focus). A longitudinal within-person intervention design is used, combined with a cross-sectional survey design. Data sources include web-based questionnaires, brief interviews, fidelity ratings, paper-and-pen questionnaires, and written documentation, collected at multiple time points with both group leaders and parents as respondents.

**Discussion:**

This project approaches fidelity and adaptation from the perspective of the professionals that manage EBIs during the sustainment phase of implementation. Although it is well known that EBIs continue to change over time, it remains to be understood how the fidelity–adaptation dilemma can be managed so that the effectiveness of interventions is retained or improved, not diluted. Moreover, the project adds to the literature by presenting an occupational health perspective on the fidelity–adaptation dilemma. It is acknowledged that fidelity and adaptation may have consequences for not only clients but also the occupational wellbeing of the professionals managing the dilemma, and subsequently, their willingness and ability to deliver EBIs in a sustainable way.

**Supplementary Information:**

The online version contains supplementary material available at 10.1186/s43058-021-00131-y.

Contributions to the literature

- In-depth analysis of how professionals manage the fidelity–adaptation dilemma during the sustainment phase of evidence-based intervention implementation.
- Investigates how the fidelity–adaptation dilemma affects professionals’ psychosocial working conditions.
- Explores how professionals can be supported in the management of fidelity and adaptation.

## Background

Evidence-based interventions (EBIs) are widely recognized as effective tools for improving individuals’ health and wellbeing. To be considered “evidence-based,” an intervention must go through rigorous evaluations to prove it is effective, often in a series of trials in both controlled (i.e., efficacy trials) and real settings (i.e., effectiveness trials). The idea behind this process is that if an intervention can show positive effects even in an effectiveness trial, then the intervention is ready to be disseminated and implemented without major adaptations.

Contrary to these expectations, adaptations to EBIs are common. Between 44 and 88% of EBI users (e.g., health professionals) have reported modifying some part of the original intervention, such as the procedure, dosage, content, format, or target group [1–3]. These adaptations are often motivated by differences between the context for which the EBIs were designed and evaluated and the context in which they are being used, leading to a misfit between the EBI and the context of its application that must be addressed [4, 5].

Although there are studies showing high fidelity to be related to better outcomes than low fidelity [6, 7], other studies have suggested that adapted EBIs may be more effective than non-adapted EBIs [8, 9]. This tension has been referred to as the fidelity–adaptation dilemma. This implies that it is a matter of either–or. Yet, contemporary conceptualizations tend to emphasize that fidelity and adaptations can coexist; that is, that the relationship is both–and, not either–or [10–12]. This makes fidelity and adaptation a paradox rather than a dilemma [13].

Reconciling this paradox entails considering how fidelity and adaptation should be managed so that the EBI can fit a specific context while retaining its core components [14, 15]. This ensures that the adapted EBI is at least non-inferior to and possibly better than the original version [16, 17]. This can be achieved either by adapting the context by removing obstacles to high fidelity or by adapting the EBI to fit the context, for example, by omitting or modifying components that are not applicable or feasible in a specific context [14]. Moreover, in order to ensure thoughtful decisions that promote fit without threatening the integrity of the EBI, adaptation decisions should be made proactively and with careful consideration of how they affect the EBI’s core components and subsequent effectiveness [1, 2, 18–20]. This implies a structured process involving multiple stakeholders and experts on the EBI, implementation, and the local context [1, 2, 18–20].

Several models and frameworks have been developed to structure and support the process of making decisions regarding adaptations [1, 2, 20]. The purpose is often to enable the adoption of an EBI by facilitating the dissemination of EBIs in settings that may differ from the EBI’s original development context. The models consist of elaborate processes involving multiple steps, including identification of mismatches that call for adaptations and pilot-testing and evaluation of the adapted version [21]. Thus, the adaptation process spans all implementation stages (i.e., exploration, preparation, implementation, and sustainment) [22]. For example, during the exploration and preparation phases, it is proposed that multiple stakeholders are engaged in a structured, rational decision process to identify obstacles to implementation that affect the fit between the EBI and the context (e.g., [2, 23]). During the active implementation phase, the models might propose systems to monitor and fidelity (and adaptation) data, enabling data-driven decisions [24]. In this phase, substantive implementation support may be available to support iterative troubleshooting processes where fidelity and adaptation decisions are part of continuous improvement cycles [25, 26].

However, less attention has been paid to how fidelity and adaptation decisions play out later in the implementation process (i.e., the sustainment phase) when the EBI has become part of everyday practice (i.e., institutionalized). In the best-case scenario, there is no additional need for decisions about fidelity and adaptation during the sustainment phase because tensions have already been resolved. However, this assumption is not upheld if the context changes, as this may throw off any carefully negotiated fit between an EBI and a context. Such an approach is illustrated in the dynamic sustainability process [17], which points to the need for continued learning and problem solving through ongoing adaptations. It is also consistent with definitions of sustainability as the ability to continuously deliver value, rather than as the consistent delivery of an EBI [27]. Therefore, the management of fidelity and adaptation during the sustainment phase needs to be further investigated.

The territory for fidelity and adaptation management is different in the sustainment phase than in the earlier implementation phases. The extra resources allocated to the implementation have generally ended, including experts and data management support. Therefore, once an EBI has been adopted and implemented, the professionals delivering the EBI become the default decision-makers regarding fidelity and adaptation. With the additional resources to manage implementation withdrawn, professionals are likely to make decisions about fidelity and adaptations under bounded conditions characterized by lack of time, information, and think-space in combination with multiple concurrent obligations [28–30], contrary to the rational, structured approach outlined in most adaptation frameworks [2, 19, 23]. However, little is known about how professionals manage fidelity and adaptation during sustained use of EBIs, and there have been few attempts to develop ways to support them in doing so.

The way in which professionals manage the fidelity–adaptation dilemma has important implications not only for the effectiveness of EBIs but also for the professionals themselves. Having to make decisions in these far from ideal situations might burden professionals. For instance, they may find themselves in emotionally or ethically charged situations in which they want to adhere to protocol but doing so is not possible, or in which they feel that adaptations would be appropriate but feel compelled to adhere to the protocol [31, 32]. This dilemma is, therefore, likely to be perceived as a potential work-related stressor [8, 33]. Thus, health professionals might be negatively affected cognitively and emotionally by dealing with the fidelity–adaptation dilemma. This highlights the need to increase our knowledge about the fidelity–adaptation dilemma as a part of professional psychosocial working conditions and to explore how professionals’ confidence and skills in managing the dilemma can be improved. To the best of our knowledge, such research is currently missing. This project aims to fill that gap.

### Aims

In this project, we will study fidelity and adaptation during the sustainment phase of the implementation of an EBI (a parenting program), from the perspective of the professionals. The program, All Children in Focus, has been shown to have positive effects on children’s and parents’ health [34], and it is widely offered by community services in Sweden.

To address the research gaps mentioned above, the aims of this project are threefold: (1) to study how adaptation and fidelity are managed by professionals when using an EBI, (2) to study how the fidelity–adaptation dilemma affects professionals’ psychosocial working conditions, and (3) to study how a structured decision support intervention influences professionals’ management of the dilemma and their psychosocial working conditions. Thus, the project focuses on professionals’ experiences of managing fidelity and adaptations when using an EBI as part of their everyday work, including how they can be supported in their decision-making.

### Research questions

The aims are developed in the following research questions (RQ):

- RQ1) How are fidelity and adaptation managed by professionals during the (sustained) use of an EBI?
- RQ2) What consequences does the fidelity and adaptation dilemma have for professionals’ experience of their psychosocial working conditions?
- RQ3) How does a decision support intervention affect the fidelity and adaptation dilemma and its consequences?
- How can the four subquestions to RQ3 be intented?
- How does the decision support function, and how is it perceived by professionals?
- How does the decision support affect professionals’ psychosocial working conditions?
- How does the decision support affect how fidelity and adaptations are managed?
- How does the decision support affect the value created by the EBI (outcomes for parents, for professionals, and for the organization)?

### Theoretical framework

The formulation of research questions and the interpretation of results are guided by the recently proposed value equation framework [14]. This theoretical framework proposes a way in which the fidelity–adaptation dilemma can be reconciled by focusing on the value that an EBI can produce rather than just the intervention effects. The framework construes implementation strategies as a method to create a fit between EBIs and context, emphasizing that fit can be achieved either by adapting the intervention to the context or the context to the intervention. The optimal decision is the one that maximizes the value that can be achieved across clients, professionals, the organization, and the system. The value equation, in turn, relies on the dynamic sustainability framework [5], which emphasizes the need to assess care settings and outcomes on an ongoing basis, not just prior to implementation, as the analyses of the process and outcome data provide ample opportunities to refine and improve the intervention [5].

Furthermore, we use the job demands-resources model as the guiding theory underlying the study of the dilemma as part of the professional psychosocial work environment [35]. The theory suggests that individuals’ experiences of strain and motivation at work are a function of the cognitive, emotional, and physical demands they face and the resources that are available to deal with these demands. Having too many demands and limited resources increases the risk of strain and stress, whereas sufficient resources in relation to demands result in a motivational process. Thus, the theory suggests that using an EBI under conditions that require decisions about fidelity and adaptation may put the professional under emotional and cognitive pressure if not met by sufficient resources.

## Methods

### Study design

This study will use a longitudinal within-person intervention design to address RQ1 and RQ3 combined with a cross-sectional survey design to address RQ2. The within-person design requires that the same individuals participate in both a control condition (using the parent program as usual) and the experimental condition (using the parenting program after participating in the decision support intervention). This design is particularly useful when randomization or recruitment of a control group is unfeasible. The within-person design may, in addition, be complemented with a pretest–posttest design without a control group for professionals with whom a within-person design is not possible (e.g., because they do not run enough parent groups).

### Setting, recruitment, and participants

The study will be performed in the context of the All Children in Focus (ABC) parent program in Sweden, in close collaboration with the organization providing training and support for ABC. The ABC program has been provided since 2011. The target group of the study is professionals (group leaders) who lead the parenting program. Parents in the groups will also be invited to participate.

The group leaders will be recruited to the study through the collaboration organization. Group leaders are from various backgrounds (e.g., teachers, psychologists, social workers); work in community organizations, such as social services and pre-schools; and are primarily from the capital region. All group leaders (*N* ≅ 800) who have received training in ABC by the organization  will be invited to participate in the study through their mailing list. After receiving written information about the aim of the project and a description of what participation entails, how integrity will be protected, and how all participation is voluntary and can be withdrawn at any time, the group leaders will be asked to provide informed consent and their contact information through an online form.

Parents will be recruited by the group leaders participating in the study. They will be introduced to the project through a video presented at the beginning of the first parent group session or by the group leaders. Parents will also receive information about the aim of the project and a description of what participation entails, how integrity will be protected, and how all participation is voluntary and can be withdrawn at any time. They will then be asked to provide their informed consent.

### The parenting program

The ABC is a universal health-promoting parenting program developed for parents with children in the age of 3–12 years old. It targets the parent–child relationship and parental everyday experience with the aim to promote children´s development [34]. The program consists of four 2.5-h group meetings in groups of up to 14 participants and one follow-up session. The program is provided in several languages, and parents can choose to attend singly or in couples. It has previously been evaluated in an RCT and shown to be efficacious [34] and cost-effective [36] (for further details, see [37]).

### The decision support intervention

The structured decision support targets the group leaders with the following core functions:

1. To provide group leaders with knowledge and awareness of the relationship between how EBIs are used and the value they produce for clients, professionals, organizations, and systems, in keeping with the value equation framework [14];
2. To enable group leaders to make informed choices concerning the adaptation of EBI; that is, to maximize the value that the EBI can produce by ensuring optimizing adherence to core components, making changes in the context if needed, and adaptations of the EBI if needed to improve use and functioning.

The decision support intervention is based on the Planned Adaptation Model [2] and the Useful Evidence Model [13] and guided by the value equation framework [14]. Participants will be guided through a process of making decisions about fidelity and adaptation based on the identification of intervention and contextual components that combine to produce the aspired outcome: value. This includes the identification of core components and the activities needed to retain them, as well as the identification of the components of the context that are non-compatible with the achievement of the aspired values.

The decision support intervention will be delivered in two 2.5-h workshops held 2–3 weeks apart. It will be conducted by the researchers and held either face-to-face or digitally, if needed due to pandemic restrictions.

The decision support intervention was developed in conjunction with an ongoing project aiming to support fidelity and adaptation decisions during earlier phases of implementation [38]. The face-to-face version has been pilot-tested as part of that project. The digital version has been tested separately with (a) staff from the collaboration organization and (b) four professionals delivering the parent program. Additional file 1 presents the Template for Intervention Description and Replication (TIDieR) checklist [39].

### Data collection procedure

**Fig. 1:**
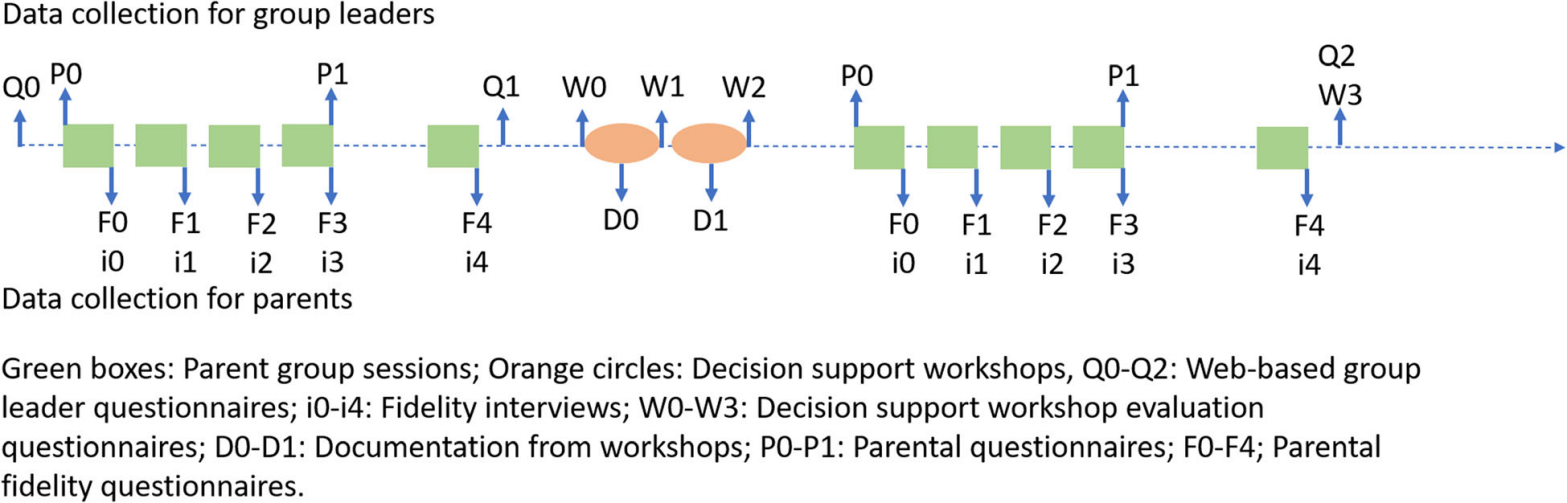
Overview of data collections

Multiple data sources will be used to address the RQs, collected at multiple time points with both group leaders and parents as respondents. We will use web-based questionnaires, brief interviews, fidelity ratings, paper-and-pen questionnaires, and written documentation (for the type of data collection methods and timing, see Fig. 1).

The group leaders will first be invited to respond to a web-based group leader questionnaire (Q0), which will include an invitation to participate in the decision support intervention. Thus, the questionnaire will be used for the cross-sectional study to address RQ2. It will also be used as a baseline for the decision support intervention (RQ3). Those agreeing to participate in the intervention will also be invited to further data collections, as outlined in Fig. 1. The web-based group leader questionnaire (Q0) will be repeated at two time points (Q1 and Q2). Additional data will be collected during the parent group sessions (with parents and group leaders as respondents) and the decision support workshops (with group leaders) (see Fig. 1).

Three sources of data will be collected in conjunction with the parent group sessions. First, a parental questionnaire (P0 and P1 in Fig. 1) will be distributed to parents during the first and last parent group sessions and used to evaluate the effect of the parent groups on parent and child behavior. Second, parents will be asked to provide fidelity ratings in a fidelity questionnaire (F0–F4) at the end of each parent group session. Group leaders will distribute questionnaires to parents. Third, brief telephone interviews will be held with the group leaders after each parent group session (i1–i4) to assess fidelity and adaptations. In line with the within-person design, the data collection will be repeated for a second group after the group leader has participated in the decision support workshops.

Two data collections will take place in conjunction with the decision support workshops. First, documentation (D0 and D1) produced by the participating group leaders during the sessions describing the fidelity–adaptation decision-making process will be copied and compiled. Second, a brief process evaluation questionnaire, including appraisals of the workshop as well as knowledge of and attitudes towards fidelity and adaptations, will be collected at four time points: before the first workshop (W0), after the first (W1) and second (W2) workshops, and at a follow-up (W3) in conjunction with the last web-based group leader questionnaire (Q2).

### Study variables and instruments

Table 1 presents the study variables and instruments for the data collection.

**Table 1:** Overview of study variables and instruments used in the data collection

Variables	Instruments/specification	Respondent	Data source and source
**Acceptability**	Acceptability of Intervention Measure—AIM [40]	Group leaders	Web-based group leader questionnaire: Q0, Q1, Q2
**Appropriateness**	Intervention Appropriateness Measure—IAM [40]	Group leaders	Web-based group leader questionnaire: Q0, Q1, Q2
**Feasibility**	Feasibility of Intervention Measure—FIM [40]	Group leaders	Web-based group leader questionnaire: Q0, Q1, Q2
**Fidelity to and adaptation of EBI**	Perceived value of ABC Perceived core components of ABC Perceived non-compatible context elements Adapted version of ABC	Group leaders	Documentation from workshop: D0, D1
	Fidelity–adaptation decisions and actions (who, how, when, what, why) [41]	Group leaders	Fidelity interviews: i0, i1, i2, i3, i4
	Reason for adaptations (reactive/proactive adaptations, logistical/philosophical, valence) [42] Type of adaptations made (e.g., tailoring, removing, lengthening, integrating, skipping elements) [43] Attitudes towards and perceived knowledge about fidelity and adaptations adapted from Determinants of Implementation Behavior Questionnaire (DIBQ) [44]	Group leaders	Web-based group leader questionnaire: Q0, Q1, Q2 Web-based group leader questionnaire: Q0, Q1, Q2 Workshop evaluation questionnaires: W0, W1, W2
	Program-specific fidelity assessment for parents (six items about how group leaders managed the session, e.g., “My group leaders focused the discussion on the session’s theme”). Developed by the developers of ABC	Parents	Fidelity questionnaire: F0, F1, F2, F3, F4
**Timeliness** [45]	Waiting time before program access	Parents	Parental questionnaire: P0
**Equity** [45]	Specific reasons for adaptations to improve equity	Group leaders	Fidelity interviews: i0, i1, i2, i3, i4
**Effectiveness**	Program-specific assessment of child and parent interaction Parenting outcomes pertaining to child and parent interaction [46], Strengths and Difficulties Questionnaire (SDQ) [47], Therapy Attitude Inventory [48]	Parents	Parental questionnaire: P0, P1
**Service-user centeredness** [45]	Structured questions in interviews about adaptations	Group leaders	Fidelity interviews: i0, i1, i2, i3, i4
**Parent satisfaction**	Client Satisfaction Scale (CSQ) [49]	Parents	Parental questionnaire: P1
**Variables pertaining to the work context of group leaders**	Implementation climate [50] Physical and social opportunity (environmental context and resources and social influences) for using the program (six items adapted from DIBQ) [44] Psychosocial work environment (work demands, influence, learning opportunities, job satisfaction, role conflict, social support). Items from Copenhagen Psychosocial Questionnaire version II (COPSOQ II) [51, 52] Perceived illegitimate tasks [53]	Group leaders	Web-based group leader questionnaire: Q0, Q1, Q2
**Variables pertaining to individual characteristics of group leaders**	Attitudes to evidence-based practice [54] Professionals’ capability and motivation (knowledge, skills, beliefs about consequences, behavioral regulation and reinforcement) for using the program (11 items adapted from DIBQ) [44] Professionals’ occupational self-efficacy [55]	Group leaders	Web-based group leader questionnaire: Q0, Q1, Q2
**Decision support intervention appraisal**	Perceived relevance, quality, and usefulness of the decision support intervention (11 items adapted from the BIC evaluation) [56] Perceived impact of the decision support intervention [56]	Group leaders Group leaders	Workshop evaluation questionnaires: W1 and W2 and web-based group leader questionnaire: Q2 Workshop evaluation questionnaires: W1, W2
**Readiness for change**	Readiness for change [56]	Group leaders	Workshop evaluation questionnaires: W0
**Background variables of group leaders**	Age, gender, educational level, work experience, profession, parenting program expertise/experience	Group leaders	Web-based group leader questionnaire: Q0
**Background variables of parents**	Parents educational level, number and age of children, marital status	Parents	Parental questionnaire: P0

### Analysis

This is a multimethod study involving both qualitative and quantitative data. The qualitative data will be analyzed through content analysis. The quantitative data will be analyzed with descriptive analyses (e.g., frequencies, correlations) and more advanced analysis, such as multilevel analysis (e.g., accounting for dependencies in parental data), and mix-methods to investigate and triangulate changes over time.

## Discussion

The goal of this project is to investigate how professionals experience and manage the fidelity–adaptation dilemma during the sustainment phase of implementation and how the dilemma affects their psychosocial working conditions. Moreover, a decision support intervention, which focuses on professionals and might serve as a tool to manage the fidelity–adaptation dilemma during sustained use, is tested.

The project will contribute to the development of knowledge on the implementation of EBIs in four main ways. First, we focus on how the fidelity–adaptation dilemma plays out after the active implementation phase, when EBIs are used as part of regular services. This complements the growing literature on how adaptations are managed that primarily focuses on earlier implementation phases, i.e., during exploration, preparation, and, to some extent, active implementation. In this, we address a research gap concerning how the fidelity–adaptation dilemma plays out during the sustainment phase, a research gap that has persisted even though it is well-known that adaptations are common as EBIs are spread and used [29, 42, 57].

Second, the study adds to the current knowledge of how professionals manage fidelity–adaptation during the use of an EBI. Understanding what guides the choices professionals make when dealing with the fidelity–adaptation dilemma can contribute to the advancement of implementation science by showing what issues remain to be solved once the main implementation support is removed. The findings may also inform the design of parental programs by indicating which parts of a program are challenging for professionals to sustain.

Third, to our knowledge, little attention has been paid to how professionals themselves are affected when managing fidelity–adaptation dilemmas. The literature so far has primarily focused on the effects of the dilemma on clients (i.e., how it impacts intervention effectiveness). Subsequently, we know little about the consequences of the dilemma from an occupational health perspective. For example, a group leader that struggles with the fidelity–adaptation dilemma may experience cognitive load or emotional distress. In addition to the effect this may have on the professionals, it may also impact their performance as group leaders and subsequently the benefits for participating parents, as emotional distress has been shown to be inversely related to empathic skills [58], which has in turn been shown to affect the benefits for participating parents [59].

Fourth, the project will complement the literature on how decisions on fidelity and adaptation can be supported by testing a structured decision support intervention, which focuses on professionals who are already using an EBI in daily practice (i.e., during sustainment). The intervention is novel in its goal of targeting how professionals make decisions, aiming to provide them with the awareness, knowledge, and skills to make decisions based on how the decision might impact the value of the EBI. The decision support intervention is the first attempt to develop and test an intervention for the sustainment phase of implementation based on the newly proposed value equation, offering a theoretical ground for fidelity–adaptation decisions [14]. In this way, the decision support intervention provides a practical tool for how professionals can be supported in considering the impact their decisions can have on the value that the EBI can result in.

## Supplementary Information

**Additional file 1.** TIDieR checklist

## Data Availability

The datasets used will be available from the corresponding author on reasonable request.

## Abbreviation

EBI: Evidenced-based intervention
