# Supplementary material for: Professionals’ management of the fidelity–adaptation dilemma in the use of evidence-based interventions—an intervention study
Source: Implement Sci Commun. 2021 Mar 16;2:31. doi: 10.1186/s43058-021-00131-y (PMC7962232; doi:10.1186/s43058-021-00131-y)
Supplement: Supplementary file 1 — Additional file 1. TIDieR checklist [file 43058_2021_131_MOESM1_ESM.pdf]

TIDieR

The Decision Support Intervention

Why:

The structured decision support targets the group leaders with the following core functions:
1) to provide group leaders with knowledge and awareness of the relationship between how EBIs are used and the value they produce for clients, professionals, organizations, and systems, in keeping with the value equation framework (1).

2) to enable group leaders to make informed choices concerning the adaptation of EBI; that is, to maximize the value that the EBI can produce by ensuring optimizing adherence to core components, making changes in the context if needed, and adaptations of the EBI if needed to improve use and functioning.

This decision support intervention focus specifically on the sustainment phase and was developed in conjunction with an ongoing project aiming to support fidelity and adaptation decisions during earlier phases of implementation (2).

1. von Thiele Schwarz U, Aarons GA, Hasson H. The Value Equation: Three complementary propositions for reconciling fidelity and adaptation in evidence-based practice implementation. BMC health services research. 2019;19(1):868.
2. Hasson H, Gröndal H, Rundgren ÅH, Avby G, Uvhagen H, von Thiele Schwarz U. How can evidence-based interventions give the best value for users in social services? Balance between adherence and adaptations: A study protocol. Implementation Science Communications. 2020;1(1):1-9.

What (material):

The decision support intervention is a structured educational activity. There is a power-point presentation that guides the user though the process. The participants receive a handout of the presentation which also includes a template that they can use to document their work and conclusions. There is also a tracking-sheet to gather information about adaptations and fidelity. The material is currently in Swedish and can be requested from the corresponding author.

What (procedures):

Participants will be guided through a process of making decisions about fidelity and adaptation based on the identification of intervention and contextual components that combine to produce the aspired outcome: value. This includes the identification of core components and the activities needed to retain them, as well as the identification of the components of the context that are non-compatible with the achievement of the aspired values. This means that the participant first explicate core components of the intervention as it was originally designed as well as outlines the core characteristics of the context where it has previously been used and the implementation strategies previously employed and the e ects associated with the intervention. They then use this to illuminate di erences to their own situation.

Who provided:

Delivering the intervention requires knowledge about implementation in and use of evidence-based interventions in practice in general and the value equation in particular. Facilitation skills are also needed. In this study the decision support is delivered by researchers with backgrounds in psychology and public health, respectively

How (mode of delivery; individual or group):

The decision support intervention will be held in groups of about 4-10 people, and either face-to-face or digitally.

Where:

The intervention will be held in Stockholm, Sweden. It is a collaboration with an agency responsible for a train-the-trainer program focusing on parenting support programs.

When and how much:

The intervention consist of 2.5-hour workshops held 2–3 weeks apart.

Tailoring: We will do a careful process evaluation of the decision support intervention in order to capture both adaptations and fidelity to the decision support intervention.

How well (planned): We will do a careful process evaluation of the decision support intervention in order to capture both adaptations and fidelity to the decision support intervention.
